# The Impact of the Quality of Care for Adults with Acute Asthma in the Emergency Department of a Tertiary Hospital: A 1-Year Follow-Up Study

**DOI:** 10.3390/clinpract15070116

**Published:** 2025-06-24

**Authors:** Carlos Martinez Rivera, Agnes Hernandez Biette, Anna Núñez Condominas, Ignasi Garcia Olive, María Basagaña Torrentó, Clara Padró Casas, Leandro Tapia Barredo, Antoni Rosell Gratacós

**Affiliations:** 1 Pneumology Department, Hospital Universitari Germans Trias i Pujol, Universitat Autònoma de Barcelona, 08916 Badalona, Spain; amhernandezb.germanstrias@gencat.cat (A.H.B.); annunez@bellvitgehospital.cat (A.N.C.); igarcia.germanstrias@gencat.cat (I.G.O.); ltapiab.germanstrias@gencat.cat (L.T.B.); arosellg.germanstrias@gencat.cat (A.R.G.); 2 Allergology Section, Hospital Universitari Germans Trias i Pujol, Universitat Autònoma de Barcelona, 08916 Badalona, Spain; mbasagana.germanstrias@gencat.cat (M.B.T.); cpadro.germanstrias@gencat.cat (C.P.C.)

**Keywords:** asthma, exacerbation, emergency medical services, discharge reports

## Abstract

**Background/Objectives**: This study evaluates the adherence to guidelines for the management of asthma exacerbations in the ED, recommendations at discharge, and impact at a 1-year of follow-up. **Methods**: An observational study of 87 asthma patients who attended the ED during 2022 and were discharged within 24 h was carried out. Data before the ED admission, care in the ED, and discharge reports, as well as the clinical characteristics at follow-up, were recorded. The relationship between complete ED discharge reports and outcome at 1 year, and factors associated with new exacerbations were analyzed. **Results**: The mean age was 51 years, 80% of the patients were women, and 50% had severe asthma. Prior to ED admission, 58.8% of patients used ICS-LABA, 26.2% triple therapy, 31.8% had not been treated, and 51.2% had presented at least one exacerbation. On ED admission, PEF was measured in 21% of patients only, decreasing to 6.8% at 3 h. In the ED discharge reports, the use of systemic corticosteroids was recommended in 76.5% of the cases and ICS-LABA in 46.9%. However, complete ED discharge reports were recorded for only 18.2% of patients. A total of 6.7% of patients were referred to a primary care physician and 29.9% to a pneumologist. Complete ED discharge forms did not improve asthma control at follow-up or reduce new exacerbations. Exacerbations before ED admission (OR 2.49, 95% CI 1.47–4.22, *p* = 0.001) and the use of any asthma controller treatment (OR 1.84, 95% CI 1.84–507, *p* = 0.017) were associated with ≥2 exacerbations at follow-up. **Conclusions**: Contact with ED did not improve disease control or reduce exacerbations. It is necessary to optimize care before, during, and after exacerbations by developing integrated programs with primary care to improve asthma management.

## 1. Introduction

Acute asthma (asthma attack or acute exacerbation) is defined as an episode of rapidly progressing worsening of dyspnea, cough, wheezing, and/or chest tightness, accompanied by a decrease in expiratory airflow that can be quantified by the measurement of lung function [1,2]. About 20% of patients with asthma require admission to an emergency department (ED), but this minority of asthmatics is responsible for more than 80% of direct healthcare costs associated with exacerbations [3]. Exacerbations in asthma are still at risk of severe complications and may result in a more rapid decline in lung function [4,5]. The global burden of exacerbations and day-to-day symptoms has increased by almost 30% in the last 20 years [6].

Appropriate urgent treatment of acute asthma remains a challenge in daily practice, with correction of hypoxemia and rapid reversal of airflow obstruction as the main goals of treatment [1,2]. Effective management requires close monitoring of symptoms and airflow obstruction, together with an adequate history and physical examination. The main treatment recommended by clinical practice guidelines [1,2] includes repeated administration of inhaled short-acting β2-agonists (SABA), early introduction of systemic glucocorticoids, and supplemental oxygen. Before discharge, a minimum educational intervention is necessary, including a confirmation of the adequacy of inhaler technique, provision of an effective written action plan, and referral to the primary care physician within 5 days, and, if necessary, to a specialist within one month. Guidelines offer evidence-based recommendations to achieve adequate control of the disease, thus reducing symptoms, hospitalizations, and relapses, but in daily practice an adequate adherence to these recommendations is frequently lacking.

Different studies carried out in the emergency setting have shown that the quality of acute asthma care is often suboptimal, including deficiencies in the assessment of asthma severity level, inadequacy of the therapeutic approach, and shortcomings in the follow-up and referral programs upon discharge [7,8,9,10]. Moreover, regardless of treatment at hospital discharge, numerous risk factors for poor outcomes of acute asthma attacks have been identified, including multimorbidity, asthma severity, poor asthma control, airflow obstruction, higher sputum eosinophils, a very high-T2 inflammatory pattern, older age, absence of a previous asthma diagnosis, uncontrolled disease, and concomitant chronic obstructive pulmonary disease (COPD) [11,12]. In order to unify practices for the care of acute asthma patients in EDs, a Spanish expert consensus group developed specific algorithms for the management of asthma exacerbations and recommendations for therapeutic strategies after discharge [13]. Although appropriate ED patient discharge plans are critical to ensure suitable education, medication, and follow-up to improve self-management and reduce subsequent hospital admissions, it has been shown that adherence to asthma discharge bundles from the ED is poor [14,15].

However, further research is needed regarding the effect of the adequacy and completeness of ED asthma discharge reports on the follow-up of asthma patients. The primary objective of this study was to assess compliance with clinical practice guidelines in recommendations provided at ED discharge after management of asthma exacerbations and the impact of compliant versus non-compliant ED discharge forms on the outcomes of patients at 1-year follow-up.

## 2. Materials and Methods

### 2.1. Study Design and Patients

This single-center retrospective observational study was carried out on an adult asthma population admitted to the ED of an acute tertiary care teaching hospital in Badalona, Barcelona (Spain) due to an episode of asthma exacerbation between 1 January and 31 December 2022. Data collection and the 1-year follow-up of patients were conducted retrospectively. The inclusion criteria were an age of 18 years or older, a diagnosis of asthma according to the criteria of the Global Initiative for Asthma (GINA) [1] established at least 12 months before inclusion in the study, a confirmed diagnosis of an acute asthma attack as the main diagnosis, a discharge from the ED within 24 h after admission, and the availability of follow-up data over 1 year after ED admission for the index exacerbation episode. Patients with severe asthma requiring hospitalization were excluded from the study, as were those diagnosed with bronchiectasis, cystic fibrosis, or lung cancer, and patients lost to follow-up. The absence of information on the main study variables was an exclusion criterion.

The study was approved by the Ethics Committee for Clinical Research (CEIC) of the Hospital Universitari Germans Trias i Pujol (Badalona, Barcelona, Spain) (code PI-23-264, approval date 18 July 2024). Written informed consent was obtained for access to the patients’ clinical data.

### 2.2. Study Outcomes

The primary outcome of this study was to assess the adequacy of a patient’s ED discharge information, i.e., compliance with the Spanish Guideline on the Management of Asthma (GEMA) [2], based on whether treatment with systemic corticosteroids and a combination of inhaled corticosteroids (ICS) and long-acting β2-agonists (LABA) was recommended, as well as whether the ED discharge report included referral to the primary care physician and the pneumologist within 7 days and 1 month after discharge, respectively. Secondary outcomes were asthma treatment prior to index exacerbation and whether the monitoring and treatment in the ED were consistent with GEMA guidelines for urgent asthma care [2].

### 2.3. Data Collection

Data were obtained from the patients’ electronic medical records, and diagnoses were selected using International Classification of Diseases (ICD-10) codes J45.21, J45.31, J45.41, J45.51, and J45.901 (mild intermittent, mild, moderate and severe persistent asthma, and unspecified asthma with acute exacerbation).

Data recorded included demographic variables, smoking history, aeroallergen sensitization, asthma severity level, the patient’s follow-up by a primary care physician and/or pneumologist prior to ED admission, asthma treatment, use of healthcare resources, and serum eosinophil count and IgE level. In relation to the index asthma exacerbation episode, the following data were collected: possible triggers, severity of asthma exacerbation, speed of onset of asthma attack, respiratory and cardiac monitoring, arterial oxygen saturation (SaO_2_), treatment administered during exacerbation, and data related to follow-up after ED discharge. Follow-up data included the number of days elapsed from discharge to primary care physician and pneumologist visits, measurement of lung function (spirometry), availability of a self-management plan, evaluation of the degree of asthma control using the Asthma Control Test (ACT) [16] (an ACT score > 20 indicates well-controlled asthma), assessment of the inhaler technique using the Test of the Adherence to Inhalers (TAI) [17], use of healthcare resources (hospitalization and ED admission), number of exacerbation episodes, and risk factors for one or more asthma attacks over 1 year after the index exacerbation episode. Poor asthma control at follow-up was defined as the presence of two or more exacerbations, which were defined as acute episodes requiring hospitalization and/or care in the ED and/or a course of systemic corticosteroids for 3 or more days.

### 2.4. Statistical Analysis

Categorical variables are expressed as frequencies and percentages, and continuous variables as mean and standard deviation (SD). The normal distribution of variables was evaluated with the Kolmogorov–Smirnov test. Patients were divided into two groups according to whether information on the ED discharge reports was adequate or inadequate (i.e., compliance with recommendations of prescribing a course of systemic corticosteroids and inhaled ICS-LABA, and follow-up instructions for referral to the primary care physician and the pneumologist) [2]. In addition, they were divided into those with less than two exacerbations and those with two or more exacerbation episodes over the follow-up period. Categorical data were compared with the chi-square test, and quantitative data with the Student *t* test or the Mann–Whitney *U* tests according to conditions of application. Multivariable logistic regression analysis was used to identify factors associated with exacerbations over the 1-year follow-up period. Clinically relevant variables with a *p* value < 0.05 in the bivariate analysis were included in the model as independent variables, with exacerbations at 1-year follow-up as the dependent variable. Odds ratio (OR), 95% confidence intervals (CIs), and the area under the receiver operating characteristic (ROC) curve (AUC) for the regression model were calculated. Statistical significance was set at *p* < 0.05. The Statistical Package for the Social Sciences (SPSS) version 22.0 (IBM Corporate, Armonk, NY, USA) was used for the analysis of data.

## 3. Results

### 3.1. Characteristics of Patients

A total of 87 patients with an episode of asthma exacerbation who met the inclusion criteria were admitted to the ED of our hospital during 2022. There were 70 women and 17 men, with a mean (SD) age of 51.2 (23) years. The main clinical characteristics of these patients are shown in Table 1. Half of the patients had severe asthma, 58.8% were currently treated with an ICS-LABA combination, and 26.2% with a triple combination of ICS-LABA and long-acting muscarinic antagonist (LAMA). Also, 30.6% did not take any controller treatment, none had received biologic agents, and 47.6% had been visited by a primary care physician and 51.7% by a pneumologist at least once. Exacerbations in the previous year were recorded in 51.2% of patients, with a mean number of exacerbations of 1.26 (1.7).

### 3.2. The Management of Exacerbations at the ED and Discharge Reports

The management of exacerbations in the ED is shown in Table 2. Exacerbations were slow-onset in 61 patients (71.8%) and had a mild severity in 50 (57.5%). Arterial oxygen saturation (SaO_2_) was measured in almost all patients (97%). However, the peak expiratory flow (PEF) was recorded initially in 17 patients only, in 7 after 1 h, and in 4 after 3 h from admission. Treatment included oxygen therapy in 50%, systemic corticosteroids in 86.7%, and starting bronchodilators and ICS in 97.6% and 38.7% of patients, respectively. Complete ED discharge reports were recorded for only 18.2% of patients, with recommendations of systemic corticosteroids in 76.5% of cases and ICS-LABA in 46.9%. Referrals to pneumologists were more frequently present (29.9%) than referrals to the primary care physician (6.7%) (Table 2).

### 3.3. Follow-Up at 1 Year

**Figure 1:**
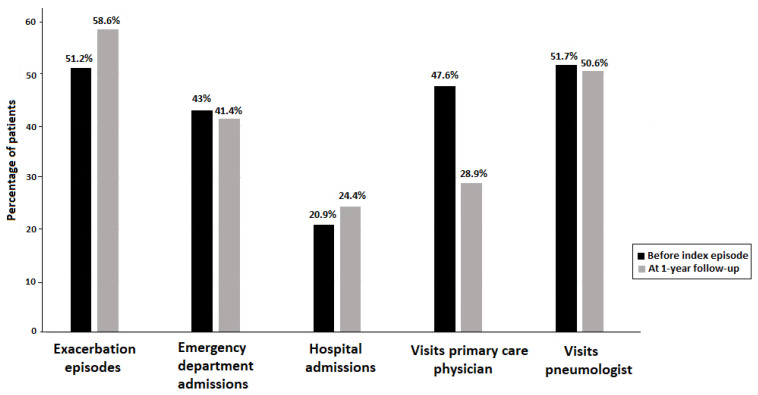
Percentages of patients with at least one asthma exacerbation episode, need of admission to the emergency department, hospital admission, and visits to the primary care physician and the pneumologist before and after 1 year of the index acute asthma attack.

During the follow-up period (Table 3), lung function tests were performed in only 41% of patients, and 38.2% had a self-management plan. The ACT and TAI questionnaires were used in 35.4% and 32.9% of patients, respectively. More than half of the patients (58.6%) suffered from at least one exacerbation episode, 41.4% required admission to the ED, and 24.4% required admission for inpatient care. Only 28.9% were visited by a primary care physician and 50.6% by a pneumologist. However, a total of 27 out of 85 patients (31.7%) were never visited by a physician and only 9 out of 83 (10.8%) were visited by both a pneumologist and a primary care physician. As shown in Figure 1, the percentages of patients with at least one exacerbation, hospitalization, and visits to the primary care physician and the pneumologist before and after 1 year of the index asthma attack were similar. The percentage of patients visited by a primary care physician decreased from 47.6% to 28.9%.

### 3.4. Risk Factors for Poor Asthma Control

Data related to the quality of ED discharge reports were recorded for 77 of the 87 patients, with complete reports for 14 and incomplete for the remaining 63. There were statistically significant differences between complete and incomplete discharge reports in the percentage of females (100% vs. 76.2%, *p* = 0.042), never smokers (46.2% vs. 72.1%, *p* = 0.030), previous visit by a pulmonologist (71.4% vs. 41.3%, *p* = 0.041), moderate/severe exacerbation (78.6% vs. 31.7%, *p* = 0.005), administration of magnesium sulfate in the ED (14.3% vs. 0%, *p* = 0.003), follow-up by a pneumologist (85.7% vs. 40.3%), performance of pulmonary function tests (71.4% vs. 31.1%, *p* = 0.005), presence of a self-management plan (69.2% vs. 33.3%, *p* = 0.017), and use of ACT (71.4% vs. 27.6%, *p* = 0.002), and TAI (57.1% vs. 27.6%, *p* = 0.035) questionnaires. The comparison of other variables prior to ED admission and at follow-up did not show significant differences (Table S1 of the Supplementary Material).

At follow-up, 24 patients presented two or more exacerbations, and 63 presented fewer than two. The distribution of study variables before the index episode in these two groups of patients is shown in Table 4. Patients with fewer than two exacerbations as compared to those with two or more exacerbations showed a lower percentage of diabetes as comorbid disease, and treatment with ICS, triple therapy, SABA on demand, visits to the pneumologist, need of hospitalization and visits to the ED, and number of exacerbations. In addition, there were significantly higher percentages of patients with mild asthma and without previous asthma treatment.

In the analysis of variables collected during ED admission for the care of the index episode and over the 1-year follow-up (Table 5), there were significant differences in a higher percentage of patients with PEF monitoring at 3 h among patients with two or more than two exacerbations, as well as higher percentages in the use of a self-management plan, ACT and TAI questionnaires, the mean number of exacerbations, a need for hospitalization and ED admissions, and a follow-up by a pneumologist.

**Figure 2:**
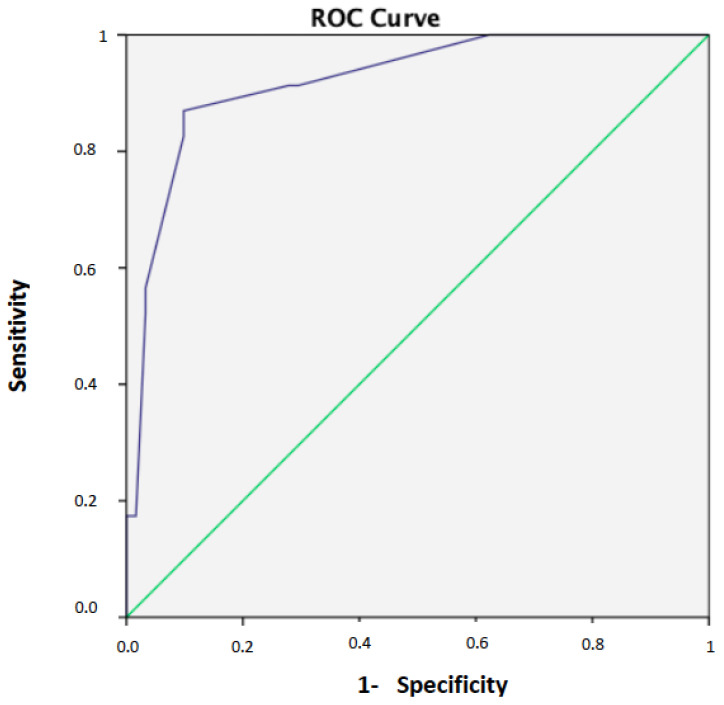
Receiver operating characteristic (ROC) curve for the model for predicting two or more exacerbations at 1-year follow-up after the index asthma exacerbation episode. The green lines represents a random classifier. The blue line represents the curve of the evaluated model.

In the logistic regression analysis, the number of exacerbations in the year prior to the index episode (OR 2.49, 95% CI 1.47–4.22, *p* = 0.001) and previous use of any asthma controller treatment (OR 1.84, 95% CI 1.84–507, *p* = 0.017) were independent factors for suffering from two or more exacerbations at follow-up. The AUC of the ROC curve was 0.921 (95% CI 0.86–0.98, *p* = 0.0001) (Figure 2).

## 4. Discussion

This study performed on a group of 87 patients with asthma exacerbation requiring care in the ED of a tertiary hospital shows that treatment recommendations and follow-up referrals included in the ED discharge reports were mostly inconsistent with the recommendations of clinical practice guidelines [2]. However, the quality of ED discharge reports was not associated with new exacerbations over the 1-year follow-up period. Previous exacerbations and being on a controller asthma medication were the only significant predictors of further exacerbations at follow-up. Also, the degree of follow-up provided in both primary care and specialized settings, both before and after the ED admission, was found to be suboptimal and could be improved.

In relation to the condition of the patients during the year prior to admission to the ED, a deficient control by primary care physicians and pneumologists was observed, with 30.6% of patients not receiving any treatment and only 58.8% being treated with an ICS-LABA combination. Notably, despite 50% having severe asthma, none of them received biologics. The fact that 51.2% had experienced previous exacerbations highlights the importance of improving the care of asthma patients in our area. Unsatisfactory asthma management and control have also been reported in other studies. The Asthma Insight and Management survey of U.S. patients and physicians conducted during 2009 for 2500 patients with asthma and 309 physicians showed that ED visits occurred once per year (60%), only 22% of patients visited a specialist for usual asthma care, and 48% had never visited a specialist [18]. In another study of 3072 patients aimed at assessing the current practice patterns of asthma care among primary care physicians in Canada, ED visits were experienced by 20% of the sample, 25% had documented evidence that they had performed spirometry, more than half had not received any form of asthma education, and only 2% received a written action plan [19]. Additionally, a study found that only 33% of 807 asthmatics who visited EDs across several Canadian hospitals were undergoing an ICS-LABA combination therapy [20]. The need for improving asthma care has been extensively recognized. The implementation of Canadian Asthma Consensus Guidelines in eight primary care practices across Ontario was associated with reductions in ED visits due to asthma from 9.9% to 5.5% and productivity loss from 12.0% to 10.3% at 12-month follow-up [21]. In addition, a group of 12 primary care global asthma experts, representing nine countries, reviewed the literature and concluded that multiple primary care interventions, including those driven by national policies, offered greater benefit than any single intervention in asthma management [22].

When analyzing the adequacy of asthma management in the ED, patients’ assessment on admission was consistent with clinical guidelines [1,2], except for PEF, which was initially measured in 21% of patients and in only 6.8% after 3 h. In a study of 323 asthmatic patients admitted to EDs of five Danish university hospitals, PEF was measured in 60% on admission, decreasing to 58% on discharge [23]. In an audit study of asthma exacerbation management in a Swiss general hospital, PEF was measured in only 14% of 160 asthma patients, and the severity should be better assessed to increase the guideline conformity [8]. Also, in an audit of the care of acute asthma in various hospitals in Wales, adherence to the British Thoracic Society (BTS)/Sign guidelines was disappointing, particularly regarding the assessment of severity and monitoring of progress [24]. Moreover, a study of 14,043 patients presenting to 201 EDs included in an audit of the Royal College of Emergency Medicine showed that standards were not met, with 25% of patients receiving nebulized β2-agonist bronchodilator within 10 min of arrival to the ED, 19% oxygen therapy, and vital signs being recorded in only 12% [25].

The ED discharge reports included all information recommended by clinical guidelines [2] for only 18.2% of patients, and the use of inhaled ICS-LABA was reported in only 47%, although it is well known that prescribing inhaled controller treatment, including ICS, prevents new acute asthma attacks [1,8]. In addition, the use of systemic corticosteroids to prevent exacerbations was present in 46.9% of the reports, and there were important deficiencies in referrals to other levels of care, especially to primary care physicians. These inconsistencies in completeness of ED discharge reports regarding asthma treatment and referrals have also been found in other studies [7,8,25].

However, an expected improvement in the control of asthma following contact with a healthcare service (i.e., ED admission) was not observed, either in the rate of exacerbations or in visits to primary care physicians and pneumologists, which in turn was independent of the quality of the ED discharge report. It is important to emphasize that 31.7% of patients were never visited by a physician over the follow-up period, which further indicates a deficiency in the care provided to these patients after having been attended in the ED due to an acute exacerbation episode.

Patients with two or more exacerbations at follow-up showed a higher number of complete discharge reports (23.8%) than those with fewer than two exacerbations (16.1%), as well as visits to the pneumologist (75% vs. 42.9%), which may suggest a higher clinical awareness of the severity of asthma potentially resulting in a greater quality of the ED discharge reports. Additionally, the delays of 17 and 76 days in consultations with the primary care physician and the pneumologists, respectively, could have been a further influential factor in exacerbations at follow-up. Despite the clinical importance of providing discharge reports following the recommendations of clinical guidelines, the impact of ED educational interventions on subsequent asthma-related outcomes remains unclear [9,26].

Previous exacerbations and requirement of inhaled controller asthma treatment were significant factors associated with new exacerbation episodes, which is consistent with a higher level of severity of asthma. Several studies have shown that an asthma attack is itself a strong predictor of an exacerbation of the disease in the future. In 763 middle-aged and older asthma patients included in the Rotterdam Study, previous exacerbations, use of SABA without concomitant controller medication, respiratory complaints, obesity, and airway obstruction were independent risk factors for exacerbations [27]. In a simple risk prediction model for asthma exacerbations using routinely collected healthcare data, older age, female gender, and prescription of more SABA and ICS in the preceding 12 months were independent factors associated with an asthma exacerbation within 3 months of the index exacerbation date [28]. SABA medications should be used cautiously, as recommended by guidelines, given that the overuse of SABA in asthma is associated with an increased risk of exacerbation and mortality [29].

Limitations of this study include the single-center characteristics and the small number of patients, probably because admission to the hospital was an exclusion criterion. The limited sample size may impact the statistical power and generalizability of findings, although the fact that the study was conducted in daily practice rather than in strict conditions of clinical trials adds clinical value to the results obtained. Difficulties in collecting more extensive asthma-related information within the year before ED admission and at the time of exacerbation itself (e.g., monitoring and treatment details during ED admission) are also limitations of the study. Inadequate documentation of medication regimens, asthma education, action plans, referrals, and exacerbating factors in hospitalized patients has also been reported in other studies [30]. Strengths of this study include the analysis of all consecutive patients with asthma exacerbation who attended the ED of a tertiary care hospital over one year and the impact of care and adequacy of ED discharge reports on the occurrence of further exacerbations at follow-up. As far as we are aware, an overall perspective of asthma patients before and during ED admission for exacerbations and the impact of ED care at follow-up have not been previously reported.

## 5. Conclusions

This real-world study shows that admission to the ED of patients with an acute exacerbation episode was not associated with a better control of asthma and did not prevent further exacerbations. Therefore, there is a need for improving the provision of care by the healthcare system for patients attending EDs due to an acute asthma attack, both before and following the index exacerbation episode. Practical implications of the study indicate that there is an urgent need to raise awareness among ED doctors to provide full instructions in compliance with guidelines on asthma management in all respects, including asthma medication and control of the disease by healthcare professionals. The approach and coordination of asthma patients at the different levels of care also need to be improved. The present findings open the door to the importance of establishing combined programs for the care of patients with asthma, particularly at the time of an acute exacerbation when requiring admission to the ED.

## Figures and Tables

**Table 1:** The clinical characteristics of 87 patients with an asthma attack.

Study Variables	Patients (%)	Mean (SD)
Female patients, n = 87	70 (80.5)	
Age, years, n = 87		51.2 (23)
Smoking status, n = 84		
Active smoker	15 (17.9)	
Ex-smoker	11 (13.1)	
Never smoker	58 (69.0)	
Comorbidity		
Obesity, body mass index (BMI) > 30 kg/m^2^, n = 83	31 (37.3)	
Hypertension, n = 87	28 (32.2)	
Diabetes mellitus, n = 87	14 (16.1)	
Gastroesophageal reflux disease (GERD), n = 82	15 (18.3)	
Nasal polyposis, n = 72	7 (9.7)	
Anxiety, n = 85	29 (34.1)	
Depression, n = 84	20 (23.8)	
Prick test, n = 87		
Positive	30 (34.5)	
Negative	7 (8.0)	
Unavailable data	50 (57.5)	
Severity of asthma, n = 54		
Mild	15 (27.8)	
Moderate	12 (22.2)	
Severe	27 (50.0)	
Previous treatment for asthma		
Inhaled corticosteroids (ICS), n = 85	56 (65.9)	
ICS, µg budesonide equivalent		471.6 (315)
ICS only, n = 85	6 (7.1)	
ICS-LABA, n = 85	50 (58.8)	
Triple therapy (ICS-LABA-LAMA), n = 84	22 (26.2)	
SABA on demand, n = 86	65 (75.6)	
Puffs of SABA, n = 86		2.7 (1.6)
No previous treatment, n = 85	26 (30.6)	
Visit to the primary care physician, n = 82	39 (47.6)	
Visit to the pneumologist, n = 87	45 (51.7)	
Need of hospitalization, n = 86	18 (20.9)	0.26 (0.5)
Ned of ED admission, n = 86	37 (43.0)	0.7 (1)
Exacerbation episodes, n = 86	44 (51.2)	1.26 (1.7)
Blood eosinophil count, cells/µL, n = 8		340 (290)
Serum IgE level, kU/L, n = 87		387 (461)

SD: standard deviation; n = number of patients in which data was recorded; LABA: long-acting β2-agonist; LAMA: long-acting muscarinic antagonist; SABA: short-acting β2-agonist; ED: emergency department.

**Table 2:** The management of the exacerbation episode and ED discharge reports in the study population of 87 patients.

Study Variables	Number of Patients (%)
Severity of exacerbation, n = 87	
Mild	50 (57.5)
Moderate	31 (35.6)
Onset of symptoms, n = 85	
Rapid onset	24 (28.2)
Slow onset	61 (71.8)
Sputum analysis, n = 87	9 (10.3)
Assessment of the cause of exacerbation, n = 86	22 (25.5)
Arterial oxygen saturation (SaO_2_), n = 87	84 (96.6)
Peak expiratory flow (PEF), n = 81	17 (21.0)
PEF monitored at 1 h, n = 59	7 (11.9)
PEF monitored at 3 h, n = 59	4 (6.8)
Treatment	
Systemic corticosteroids, n = 83	72 (86.7)
Oxygen therapy, n = 80	40 (50.0)
Magnesium sulfate, n = 83	2 (2.4)
Inhaled bronchodilators, n = 83	81 (97.6)
Inhaled corticosteroids (ICS), n = 75	29 (38.7)
ED discharge report	
Use of systemic corticosteroids, n = 81	62 (76.5)
Use of inhaled ICS-LABA, n = 81	38 (46.9)
Reports with treatment variables only, n = 81	30 (37.0)
Referral to the primary care physician, n = 75	5 (6.7)
Referral to a pneumologist, n = 77	23 (29.9)
Complete (compliant) report, n = 77	14 (18.2)

ED: emergency department; n: number of patients in which data was recorded; LABA: long-acting β2-agonist.

**Table 3:** Variables related to the follow-up at 1 year after discharge from the ED in the study population of 87 patients.

Study Variables	Patients (%)	Mean (SD)
Lung function tests, n = 83	34 (41.0)	
Self-management plan, n = 76	29 (38.2)	
Asthma Control Test (ACT), n = 79	28 (35.4)	
Test of Adherence to Inhalers (TAI), n = 79	26 (32.9)	
Exacerbation episodes, n = 87		
At least one episode	51 (58.6)	
Number of episodes		1.25 (1.7)
Need for inpatient care, n = 86	21 (24.4)	
Number of hospitalizations		0.36 (0.7)
Need for ED admission, n = 87	36 (41.4)	
Number of ED admissions		0.63 (1)
Follow-up by a primary care physician, n = 83	24 (28.9)	
Days before a visit with the primary care physician		16.7 (19)
Follow-up by a pneumologist, n = 85	43 (50.6)	
Days before a visit with the pneumologist		75.5 (72)

ED: emergency department; SD: standard deviation.

**Table 4:** The distribution of study variables recorded before admission to the ED according to the number of exacerbations at follow-up.

Study Variables	Exacerbation Episodes		
	<2 (n = 63)	≥2 (n = 24)	*p* Value
Female patients, n/total (%)	50/63 (79.4)	20/24 (83.3)	0.667
Age, years, mean (SD)	51.1 (22.2)	54 (23.9)	0.490
Never smoker, n/total (%)	43/60 (71.7)	15/24 (62.5)	0.112
Comorbidity, n/total (%)			
Obesity, BMI > 30 kg/m^2^	21/59 (35.6)	10/24 (41.7)	0.604
Hypertension	21/63 (33.3)	7/24 (29.2)	0.710
Diabetes mellitus	7/63 (11.1)	7/17 (29.2)	0.041
Gastroesophageal reflux disease (GERD)	10/61 (16.4)	5/21 (23.8)	0.448
Nasal polyposis	3/50 (6)	4/22 (18.2)	0.108
Anxiety	20/62 (32.3)	9/23 (39.1)	0.553
Depression	13/61 (21.3)	7/23 (30.4)	0.381
Mild asthma, n/total (%)	14/35 (40.0)	1/19 (5.3)	0.003
Previous treatment for asthma, n/total (%)			
Inhaled corticosteroids (ICS)	37/62 (59.7)	19/23 (82.6)	0.048
ICS, µg budesonide equivalent, mean (SD)	433.2 (273.3)	542.3 (380)	0.229
ICS only	4/62 (6.5)	2/23 (8.7)	0.720
ICS-LABA	33/62 (53.2)	18/24 (75)	0.065
Triple therapy (ICS-LABA-LAMA)	9/61 (14.8)	13/23 (56.5)	0.0001
SABA on demand	42/63 (66.7)	23/24 (95.8)	0.005
Puffs of SABA, mean (SD)	2.53 (1.28)	2.95 (1.93)	0.584
No previous treatment, n/total (%)	25/62 (40.3)	2/23 (8.7)	0.005
Visit to the primary care physician, n/total (%)	30/60 (50.0)	9/22 (40.9)	0.637
Visit to a pneumologist, n/total (%)	27/63 (42.9)	18/24 (75.0)	0.007
Need of hospitalization, n/total (%)	8/62 (12.9)	10/24 (41.7)	0.003
Number of hospitalizations, mean (SD)	0.15 (0.4)	0.54 (0.8)	0.025
Ned of ED admission, n/total (%)	16/62 (25.8)	21/24 (87.5)	0.0001
Number of ED admissions, mean (SD)	0.31 (0.6)	1.75 (1.2)	0.0001
Exacerbation episodes, n/total (%)	22/62 (35.5)	22/24 (91.7)	0.0001
Number of exacerbations, mean (SD)	0.60 (1.1)	2.96 (1.9)	0.0001
Blood eosinophil count, cells/µL, mean (SD)	354 (280)	301 (505)	0.491
Serum IgE level, kU/L, mean (SD)	428 (441)	341 (505)	0.692

ED: emergency department; SD: standard deviation; LABA: long-acting β2-agonist; LAMA: long-acting muscarinic antagonist; SABA: short-acting β2-agonist.

**Table 5:** The distribution of study variables recorded during admission to the ED and at follow-up according to the number of exacerbations over 1 year.

Study Variables	Exacerbation Episodes		
	<2 (n = 63)	≥2 (n = 24)	*p* Value
Moderate/severe exacerbation, n/total (%)	25/63 (39.6)	12/24 (50)	0.684
Slow onset of symptoms, n/total (%)	43/62 (69.4)	18/23 (78.3)	0.418
Arterial oxygen saturation (SaO_2_), n/total (%)	62/63 (98.4)	22/24 (91.7)	0.123
Peak expiratory flow (PEF), n/total (%)	9/57 (15.8)	8/24 (33.3)	0.077
PEF monitored at 1 h	6/43 (14)	1/16 (6.3)	0.416
PEF monitored at 3 h	1/42 (2.4)	3/17 (17.6)	0.035
Treatment, n/total (%)			
Systemic corticosteroids	49/59 (81.7)	23/24 (95.8)	0.241
Oxygen therapy	30/58 (51.7)	10/22 (45.5)	0.617
Magnesium sulfate	2/59 (3.4)	0/24 (0)	0.361
Inhaled bronchodilators	58/60 (96.7)	23/23 (100)	0.375
Inhaled corticosteroids (ICS)	23/57 (40.4)	6/18 (33.3)	0.594
ED discharge report, n/total (%)			
Use of systemic corticosteroids	44/58 (75.9)	18/23 (78.3)	0.818
Use of inhaled ICS-LABA	25/58 (43.1)	13/23 (56.5)	0.275
Reports with treatment variables only	19/58 (32.8)	11/23 (47.8)	0.205
Referral to the primary care physician	3/54 (5.6)	2/21 (9.5)	0.536
Referral to a pneumologist	15/56 (26.8)	8/21 (38.1)	0.334
Complete (compliant) report	9/56 (16.1)	5/21 (23.8)	0.433
Data at 1-year follow-up, n/total (%)			
Lung function tests	21/60 (35)	13/23 (56.5)	0.074
Self-management plan	15/54 (27.8)	14/22 (63.6)	0.004
Asthma Control Test (ACT)	14/57 (24.6)	14/22 (63.6)	0.001
Test of Adherence to Inhalers (TAI)	12/57 (21.1)	14/22 (63.6)	0.0001
Exacerbation episodes	27/63 (57.1)	24/24 (100)	0.0001
Number of episodes, mean (SD)	0.43 (0.5)	3.42 (1.74)	0.0001
Need for inpatient care	7/62 (11.3)	14/24 (58.3)	0.0001
Number of hospitalizations, mean (SD)	0.11 (0.32)	1 (1)	0.0001
Need for ED admission	16/63 (25.4)	20/24 (83.3)	0.0001
Number of ED admissions, mean (SD)	0.27 (0.48)	1.58 (1.35)	0.0001
Follow-up by a primary care physician	18/61 (29.5)	6/22 (27.3)	0.843
Days before a visit with the primary care physician, mean (SD)	17.4 (21.5)	14.3 (10.1)	0.923
Follow-up by a pneumologist	25/62 (40.3)	18/23 (78.3)	0.002
Days before a visit with the pneumologist, mean (SD)	87.7 (81)	58.6 (54)	0.300

ED: emergency department; SD: standard deviation.

## Data Availability

Study data are available from the corresponding author (C.R.M.) upon request.

## Supplementary Materials

The following supporting information can be downloaded at https://www.mdpi.com/article/10.3390/clinpract15070116/s1, Table S1: Variables acquired before, during, and one year after asthma exacerbation based on complete or incomplete discharge report.

## Abbreviations

The following abbreviations are used in this manuscript:

ED	Emergency department
ICS	Inhaled corticosteroids
LABA	Long-acting β2-agonists
SABA	Short-acting β2-agonists
COPD	Chronic obstructive pulmonary disease
GINA	Global Initiative for Asthma
CEIC	Ethics Committee for Clinical Research
GEMA	Guideline on the Management of Asthma
ICD	International Classification of Diseases
ACT	Asthma Control Test
TAI	Test of Adherence to Inhalers
SaO_2_	Arterial oxygen saturation
SD	Standard deviation
OR	Odds ratio
CI	Confidence interval
ROC	Receiver operating characteristic curve
AUC	Area under the ROC curve
SPSS	Statistical Package for the Social Sciences
LAMA	Long-acting muscarinic antagonist
BMI	Body mass index
GERD	Gastroesophageal reflux disease
PEF	Peak expiratory flow

## References

[1] Global Initiative for Asthma 2023 GINA Report, Global Strategy for Asthma Management and Prevention. https://ginasthma.org/2023-gina-main-report/.

[2] Plaza Moral V., Alobid I., Álvarez Rodríguez C., Blanco Aparicio M., Ferreira J., García G., Gómez-Outes A., Garín Escrivá N., Gómez Ruiz F., Hidalgo Requena A. (2023). GEMA 5.3. Spanish Guideline on the Management of Asthma. Open Respir. Arch..

[3] Dougherty R.H., Fahy J.V. (2009). Acute exacerbations of asthma: Epidemiology, biology and the exacerbation-prone phenotype. Clin. Exp. Allergy.

[4] Caballero-Segura F.J., Lopez-de-Andres A., Jimenez-Garcia R., de Miguel-Yanes J.M., Hernández-Barrera V., Carabantes-Alarcon D., Zamorano-Leon J.J., de Miguel-Díez J. (2022). Trends in asthma hospitalizations among adults in Spain: Analysis of hospital discharge data from 2011 to 2020. Respir. Med..

[5] O’Byrne P.M., Pedersen S., Lamm C.J., Tan W.C., Busse W.W., START Investigators Group (2009). Severe exacerbations and decline in lung function in asthma. Am. J. Respir. Crit. Care Med..

[6] Jackson D.J., Sykes A., Mallia P., Johnston S.L. (2011). Asthma exacerbations: Origin, effect, and prevention. J. Allergy Clin. Immunol..

[7] Rueegg M., Busch J.M., van Iperen P., Leuppi J.D., Bingisser R. (2023). Characteristics of asthma exacerbations in emergency care in Switzerland-demographics, treatment, and burden of disease in patients with asthma exacerbations presenting to an emergency department in Switzerland (CARE-S). J. Clin. Med..

[8] Schnyder D., Lüthi-Corridori G., Leuppi-Taegtmeyer A.B., Boesing M., Geigy N., Leuppi J.D. (2023). Audit of asthma exacerbation management in a Swiss general hospital. Respiration.

[9] Tapp S., Lasserson T.J., Rowe B.H. (2007). Education interventions for adults who attend the emergency room for acute asthma. Cochrane Database Syst Rev..

[10] Hasegawa K., Chiba T., Hagiwara Y., Watase H., Tsugawa Y., Brown D.F., Camargo C.A., Japanese Emergency Medicine Network Investigators (2013). Quality of care for acute asthma in emergency departments in Japan: A multicenter observational study. J. Allergy Clin. Immunol. Pract..

[11] Pola-Bibian B., Dominguez-Ortega J., Vilà-Nadal G., Entrala A., González-Cavero L., Barranco P., Cancelliere N., Díaz-Almirón M., Quirce S. (2024). Asthma exacerbations in a tertiary hospital: Clinical features, triggers, and risk factors for hospitalization. J. Investig. Allergol. Clin. Immunol..

[12] Domínguez-Ortega J., Luna-Porta J.A., Olaguibel J.M., Barranco P., Arismendi E., Barroso B., Betancor D., Bobolea I., Caballero M.L., Cárdaba B. (2023). Exacerbations among patients with asthma are largely dependent on the presence of multimorbidity. J. Investig. Allergol. Clin. Immunol..

[13] Piñera Salmerón P., Delgado Romero J., Domínguez Ortega J., Labrador Horrillo M., Álvarez Gutiérrez F.J., Martínez Moragón E., Moral V.P., Rodríguez C.Á., Franco J.M. (2018). Documento de consenso para el manejo del paciente asmático en urgencias. Emergencias.

[14] Forward C., O’Loghlin R., Mulryan H., Langan D., Harrison M., Rutherford R., Cusack R. (2022). Asthma patients discharged from the emergency department in Ireland: An unmet need?. Eur. Respir. J..

[15] Awli Y.F., Addis G. (2021). An audit of the British Thoracic Society asthma discharge care bundle in a teaching hospital. Br. J. Nurs..

[16] Jia C.E., Zhang H.P., Lv Y., Liang R., Jiang Y.Q., Powell H., Fu J.J., Wang L., Gibson P.G., Wang G. (2013). The Asthma Control Test and Asthma Control Questionnaire for assessing asthma control: Systematic review and meta-analysis. J. Allergy Clin. Immunol..

[17] Plaza V., Fernández-Rodríguez C., Melero C., Cosío B.G., Entrenas L.M., de Llano L.P., Gutiérrez-Pereyra F., Tarragona E., Palomino R., López-Viña A. (2016). Validation of the ‘Test of the Adherence to Inhalers’ (TAI) for asthma and COPD patients. J. Aerosol Med. Pulm. Drug Deliv..

[18] Murphy K.R., Meltzer E.O., Blaiss M.S., Nathan R.A., Stoloff S.W., Doherty D.E. (2012). Asthma management and control in the United States: Results of the 2009 Asthma Insight and Management survey. Allergy Asthma Proc..

[19] Tsuyuki R.T., Sin D.D., Sharpe H.M., Cowie R.L., Nilsson C., Man S.F., Alberta Strategy to Help Manage Asthma (ASTHMA) Investigators (2005). Management of asthma among community-based primary care physicians. J. Asthma.

[20] Villa-Roel C., Borgundvaag B., Majumdar S.R., Emond M., Campbell S., Sivilotti M., Abu-Laban R.B., Stiell I.G., Aaron S.D., Senthilselvan A. (2019). Reasons and outcomes for patients receiving ICS/LABA agents prior to, and one month after, emergency department presentations for acute asthma. J. Asthma.

[21] To T., Cicutto L., Degani N., McLimont S., Beyene J. (2008). Can a community evidence-based asthma care program improve clinical outcomes?: A longitudinal study. Med. Care.

[22] Fletcher M.J., Tsiligianni I., Kocks J.W.H., Cave A., Chunhua C., Sousa J.C., Román-Rodríguez M., Thomas M., Kardos P., Stonham C. (2020). Improving primary care management of asthma: Do we know what really works?. NPJ Prim. Care Respir. Med..

[23] Backer V., Harving H., Søes-Petersen U., Ulrik C.S., Plaschke P., Lange P. (2008). Treatment and evaluation of patients with acute exacerbation of asthma before and during a visit to the ER in Denmark. Clin. Respir. J..

[24] Davies B.H., Symonds P., Mankragod R.H., Morris K. (2009). A national audit of the secondary care of “acute” asthma in Wales—February 2006. Respir. Med..

[25] The Royal College of Emergency Medicine Moderate & Acute Severe Asthma. Clinical Audit 2016/17. National Report. https://rcem.ac.uk/wp-content/uploads/2021/11/Moderate_and_Acute_Severe_Asthma_Clinical_Audit_2016_17.pdf.

[26] Villa-Roel C., Nikel T., Ospina M., Voaklander B., Campbell S., Rowe B.H. (2016). Effectiveness of educational interventions to increase primary care follow-up for adults seen in the emergency department for acute asthma: A systematic review and meta-analysis. Acad. Emerg. Med..

[27] de Roos E.W., Lahousse L., Verhamme K.M.C., Braunstahl G.J., In ‘t Veen J.C.C.M., Stricker B.H., Brusselle G.G.O. (2021). Incidence and predictors of asthma exacerbations in middle-aged and older adults: The Rotterdam Study. ERJ Open Res..

[28] Kallis C., Calvo R.A., Schuller B., Quint J.K. (2023). Development of an asthma exacerbation risk prediction model for conversational use by adults in England. Pragmat. Obs. Res..

[29] Nwaru B.I., Ekström M., Hasvold P., Wiklund F., Telg G., Janson C. (2020). Overuse of short-acting β2-agonists in asthma is associated with increased risk of exacerbation and mortality: A nationwide cohort study of the global SABINA programme. Eur. Respir. J..

[30] Arnold L.B., Usery J.B., Finch C.K., Wallace J.L., Deaton P.R., Self T.H. (2009). Inadequate documentation of asthma management in hospitalized adult patients. South. Med. J..

