# Supplementary material for: The Impact of the Quality of Care for Adults with Acute Asthma in the Emergency Department of a Tertiary Hospital: A 1-Year Follow-Up Study
Source: Clin Pract. 2025 Jun 24;15(7):116. doi: 10.3390/clinpract15070116 (PMC12293119; doi:10.3390/clinpract15070116)
Supplement: Supplementary file 1 [file clinpract-15-00116-s001.zip › clinpract-3538684-supplementary.pdf]

Table S1. Variables acquired before, during and one year after asthma exacerbation based on complete or incomplete discharge report

Variables	Complete discharge report (n=14)	Incomplete discharge report (n = 63)	*p* value
Females, n/t (%)	14/14(100)	48/63(76.2)	0.042
Age, years, mean (SD)	50(18)	50.21(23.6)	0.926
Mild Asthma, n/total (%)	2/10(20)	13/39(33.3)	0.597
Ansiety, n/total (%)	6/14(42.9)	18/62(29)	0.315
Depression, n/total (%)	1/13(7.7)	16/63(25.4)	0.163
Poliposis, n/total (%)	1/13(7.7)	5/51(9.8)	0.816
Obesity, n(%)	5/14(35.7)	21/59(35.6)	0.993
ERGE , n(%)	4/14(28.6)	11/61(18)	0.374
Bronchiectasis, n (%)	3/9(33.3)	11/32(34.4)	0.954
HTA, n (%)	5/14(35.7)	18/63(28.6)	0.597
DM, n (%)	2/14(14.3)	9(63(14.3)	1
ICS, μg budesonide equivalent, mean (SD)	530(409)	435(278)	0.502
Never smoker, n(%)	6/13(46.2)	44/61(72.1)	0.030
Previous visit by GP, n(%)	4/13(30.8)	30/61(49.2)	0.227
Previous visit by PNL, n (%)	10/14(71.4)	26/63(41.3)	0.041
Previous only ICS, n(%)	0/14(0)	6/56(9.7)	0.225
Previous ICS, n(%)	9/14(64.3)	42/62(67.7)	0.804
Previous treatment ICS-LABA n(%)	9/14(64.3)	37/62(59.7)	0.750
Previous treatment triple terapy n(%)	5/14(35.7)	15/62(24.2)	0.377
Without previous treatment, n (%)	4/14(28.6)	19/62(30.6)	0.879
SABA on demand, n(%)	11/14(78.6)	47/63(74.6)	0.755
Number of puffs of SABA, mean (SD)	2.89(1.7)	2.5(1.3)	0.513
Patients requiring hospitalization n/t(%)	4/14(28.6)	14/49(23.4)	0.612
Number of hospitalizations mean (SD)	0.43(0.85)	0.25(0.51)	0.566
Patients requiring emergency admissions n/t(%)	8/14(57.1)	24/63(38.1)	0.191
Number of emergency admissions mean (SD)	0.86(0.95)	0.70(1.1)	0.312
Patients exacerbated n/t(%)	10/14(71.4)	29/63(46)	0.086
Number of total exacerbations, mean (SD)	1.71(2.3)	1.14(1.5)	0.267
Blood eosinophils (cel/microl), mean (SD)	393(256))	346(307)	0.339
Blood IgE (kU/l), mean (SD)	264(315)	443(523)	0.477

Variables	Complet discharge report (n= 14)	Incomplet discharge report (n = 63)	*p* value
Slow-onset exacerbation n/t(%)	10/14(71.4)	45/62(72.6)	0.931
Moderate/Severe exacerbation n/t (%)	11/14(78.6)	20/63(31.7)	0.005
SatpO2 obtained n/t (%)	14/14(100)	60/63(95.2)	0.405
Peakflow obtained n/t (%)	5/14(35.7)	9/57(15.8)	0.093
Corticosteroids administrated n/total (%)	13/14(92.9)	49/60(81.7)	0.578
Oxigenotherapy, n/t(%)	8/13(61.5)	25/58(43.1)	0.228
Bronchodilators administrated, n/t(%)	14/14(100)	57/59(96.6)	0.485
Magnesium Sulphatic administrated, n(%)	2/14(14.3)	0/59(0)	0.003
ICS administrated, n/t (%)	4/12(33.3)	19/54(35.2)	0.903
Peakflow monitored at 1 hour, n/t(%)	2/11(18.2))	4/41(9.8)	0.437
Peakflow monitored at 3 hour, n/t (%)	1/11(9.1)	2/42(4.8)	0.580
Final Report include GP derivation n/t(%)	1/13(7.7)	4/62(6.5)	0.870
Final Report include PNL derivation n/t(%)	14/14(100)	9/63(14.3)	0.000
Final Report include systemic corticosteroids n/t(%)	14/14(100)	44/63(69.8)	0.018
Final Report include ICS/LABA n/t(%)	14/14(100)	20/63(31.7)	0.000
Final Report only with treatment n/t(%)	14/14(100)	12/63(19)	0.000
Patients requiring hospitalization n/t(%)	4/14(28.6)	14/63(22.2)	0.612

Patients requiring emergency admissions n/t(%)	6/14(42.9)	25/63(39.7)	0.827
Patients exacerbated n/t(%)	10/14(71.4)	34/29(54)	0.232
Number hospitalizations mean (SD)	0.50(0.86)	0.30(0.66)	0.475
Number of emergency admissions, mean (SD)	0.86(1.4)	0.60(0.96)	0.728
Number of total exacerbations, mean (SD)	1.86(2.1))	1.13(1.6)	0.2670
Follow up by GP n/t (%)	3/14(21.4)	18/61(29.5)	0.544
Follow up by PNL n/t(%)	12/14(85.7)	25/62(40.3)	0.002
Days before GP visit, mean (SD)	15.33(7.5)	17.33(21.9)	0.313
Days before PNL visit, mean (SD)	48(58.8)	84.5(71)	0.071
Lung test made n/t (%)	10/14(71.4)	19/61(31.1)	0.005
Self-management plan n/t(%)	9/13(69.2)	19/57(33.3)	0.017
TAI test made n/t (%)	8/14(57.1)	16/58(27.6)	0.035
ACT test made n/t (%)	10/14(71.4)	16/58(27.6)	0.002
